# Prediction of tenderness in bovine *longissimus thoracis* et lumborum muscles using Raman spectroscopy

**DOI:** 10.5713/ab.22.0451

**Published:** 2023-02-26

**Authors:** María Sumampa Coria, María Sofía Castaño Ledesma, Jorge Raúl Gómez Rojas, Gabriela Grigioni, Gustavo Adolfo Palma, Claudio Darío Borsarelli

**Affiliations:** 1 Instituto de Bionanotecnología del NOA (INBIONATEC), CONICET, Universidad Nacional de Santiago del Estero, G4206XCP, Santiago del Estero, Argentina; 2 Universidad Nacional de Santiago del Estero. Facultad de Agronomía y Agroindustrias. Instituto para el desarrollo agropecuario del semiárido (INDEAS), G4200ABT, Santiago del Estero, Argentina; 3 Universidad de Morón. Facultad de Agronomía y Ciencias Agroalimentarias, Buenos Aires, B1708JPD, Argentina; 4 Instituto Tecnología de Alimentos - Instituto de Ciencia y Tecnología de Sistemas Alimentarios Sustentables, UEDD INTA CONICET, CP 1712 Castelar, Buenos Aires, Argentina; 5 Universidad Nacional de Santiago del Estero. Facultad de Agronomía y Agroindustrias. Instituto de Ciencias Químicas (ICQ), G4200ABT, Santiago del Estero, Argentina

**Keywords:** Chemiometric Analysis, Meat Quality, Raman Spectroscopy, Tenderness Prediction

## Abstract

**Objective:**

This study was conducted to evaluate Raman spectroscopy technique as a non-invasive tool to predict meat quality traits on Braford *longissimus thoracis et lumborum* muscle.

**Methods:**

Thirty samples of muscle from Braford steers were analyzed by classical meat quality techniques and by Raman spectroscopy with 785 nm laser excitation. Water holding capacity (WHC), intramuscular fat content (IMF), cooking loss (CL), and texture profile analysis recording hardness, cohesiveness, and chewiness were determined, along with fiber diameter and sarcomere length by scanning electron microscopy. Warner-Bratzler shear force (WBSF) analysis was used to differentiate tender and tough meat groups.

**Results:**

Higher values of cohesiveness and CL, together with lower values of WHC, IMF, and shorter sarcomere were obtained for tender meat samples than for the tougher ones. Raman spectra analysis allows tender and tough sample differentiation. The correlation between the quality attributes predicted by Raman and the physical measurements resulted in values of R^2^ = 0.69 for hardness and 0,58 for WBSF. Pearson’s correlation coefficient of hardness (r = 0.84) and WBSF (r = 0.79) parameters with the phenylalanine Raman signal at 1,003 cm^−1^, suggests that the content of this amino acid could explain the differences between samples.

**Conclusion:**

Raman spectroscopy with 785 nm laser excitation is a suitable and accurate technique to identify beef with different quality attributes.

## INTRODUCTION

Tenderness and juiciness are the most important quality traits for overall beef palatability [1]. In addition, studies show that consumers are willing to pay a higher price for beef cuts with guaranteed tenderness [2]. The best approach to assessing sensory attributes that provide the most accurate prediction of customer response is through sensory panels. However, these tests are not suitable for routine application by commercial processors as they are destructive, resource-intensive, costly, and time-consuming. Therefore, it is necessary to develop rapid, non-invasive, and non-destructive methods for the evaluation of meat quality that are suitable for use by commercial meat processors [3]. In this regard, several studies have been conducted to investigate the correlation between these sensory properties and the physical parameters measured [4–6]. Methods based on early postmortem assessment of meat quality, such as DNA or protein analysis, are accurate but are limited by the need for expensive instruments and skilled personnel.

Recent technological advances have made optical and spectroscopic methods more suitable for the commercial evaluation of meat quality [7–10]. Among them, Raman spectroscopy is a vibrational spectroscopic method that can be used to evaluate both the structure and composition of meat samples [11]. Several studies have reported on the potential of this technique in combination with chemometric analysis to predict objective meat quality traits in pork [12,13], lamb [14,15], and beef [16–19]. Previously, it was suggested that some muscles from some species may have a better prediction of meat quality traits [20]. For instance, Bauer et al [17] predicted shear strength with R^2^ = 0.33 with a 671 nm handheld Raman system in *gluteus medius* muscles of young bulls of different origin (90% Simmental and 10% mixed origin) aged for 14 days, Fowler et al [16], suggest that a 671 nm handheld Raman spectroscopic device can predict sensory tenderness with R^2^ = 0.47 in *longissimus lumborum* muscles aged 3 and 21 days. Promising results are reported in bovine *longissimus dorsi* muscles using Raman spectroscopy with 780 and 785 nm laser lines. In addition, Cama-Moncunill et al [18] predicted Warner-Bratzler shear strength with R^2^ = 0.48 in frozen *longissimus thoracis et lumborum* muscle of crossbred bull and elite Irish bovine steers using a Raman spectrometer with a 780 nm laser, and Chen et al [19] demonstrated that the portable Raman device with 785 nm laser could predict Meulle-net-Owens razor shear with R^2^ = 0.81 and toughness with R^2^ = 0.82 in 2-day-aged Chinese yellow (Yanbian) bull *longissimus dorsi* muscles.

In Argentina, as a consequence of the expansion of agriculture, British breeds were replaced by other genotypes, such as composites between *Bos indicus* and *Bos Taurus* breeds. Among them, development of the Braford breed was improved by challenging cattle production systems to meet the demand for high-quality meat. Therefore, in this research, we explore the capability of Raman spectroscopy using a 785 nm laser to assess post-mortem meat quality traits of Braford cattle *longissimus thoracis et lumborum* muscle as a simple and accurate analytical prediction tool for the meat industry.

## MATERIALS AND METHODS

### Samples

Animal handling and experimental procedures were followed according to the Animal Welfare Procedures Manual of the National Animal Health Service of Argentina (SENASA). Samples of the *longissimus thoracis et lumborum* muscles were obtained from thirty Braford (5/8 Hereford y 3/8 Brahman) steers. Animals were slaughtered when they were an average age of 26 months and 464±34 kg body weight. After slaughter and a 48-hour chilling period between 1°C and 5°C, the fillet block between the 9th and 13th rib of each left half-carcass was removed. The block was deboned, vacuum packed, and frozen at −18°C until experimental determinations. This methodology was used to carry out the analysis of the samples in a controlled and sequential way, avoiding multiple freeze-thaw cycles [3,8].

### Intramuscular fat content, water holding capacity and cooking loss determinations

All steak sections were divided into 2.5 cm thick slices, and before measurements, the slices were thawed at 4°C for 24 h.

Intramuscular fat content (IMF) was determined according to official methods [21] in two samples of 5 g, trimmed of external fat.

Water holding capacity (WHC) was determined following the filter paper press methodology [22]. Briefly, this technique consists of compressing 0.30±0.02 g of sample, using a 90 mm diameter filter paper (Munktell Filter, Quality 1003) and measuring the wetted area. The WHC was expressed as the percentage of meat-free juice expelled (WHC = meat area/total area of infiltrated liquid ×100). This procedure assumes that the area of the squeezed juice ring absorbed by the filter paper is related to the amount of meat-free water. Each sample was analyzed twice.

For cooking loss (CL) determinations, each steak was deboned, trimmed of fat and epimysium, weighed, and subjected to heat treatment with dry-heat cooking (oven temperature, 170°C; temperature of the thermal center of the samples, 71°C) [23]. The CL value was calculated as the percentage of weight loss relative to the initial weight of the sample, (equation 1), where sample *a* and sample *b* are the sample weight before and after the heat treatment. respectively.


$$\text{CL}(\%)=\frac{sample\;a-smple\;b}{sample\;a}\times 100$$

### Texture profile analysis

Texture profile analysis (TPA) and Warner-Bratzler shear force (WBSF) determinations were carried out with 2.5 cm thick steaks of cooked meat. Once thawed, meat cuts were deboned, weighed, and placed in a pre-heated electric grill until they reached a final internal temperature of 71°C. Cooked steaks were weighed and cooled down to <10°C overnight. TPA measurements were obtained by cutting 1.3 cm diameter cores from each steak parallel to the muscle fiber orientation each of which was subjected to the action of the TA.XT Plus texturometer (Stable Micro Systems Ltd, Surrey, UK) using a 10 mm in diameter cylindrical probe. The samples were then placed under the probe which moved downward at a constant speed of 3.0 mm/s (pre-test), 1.0 mm/s (test), and 3.0 mm/s (post-test). When the probe first came in contact with the sample, the software automatically recorded the thickness of the sample. The probe continued down a preset percentage of the sample thickness (75%), returned to the initial point of contact with the sample, and paused for a period of 2 s before starting the second compression cycle. Through this analysis, the parameters of hardness, cohesiveness, and chewiness were determined. Hardness is the maximum resistance during the first compression of meat, representing the hardness of the sample in the first bite [24]. Cohesiveness is the ratio (dimensionless) between the positive force during the second compression cycle and that of the first one and represents the strength of the internal bonds, which maintains the structure of a sample [25]. Finally, springiness is the degree to which the meat returns to its original height after being compressed. Therefore, the chewiness is calculated as hardness×cohesiveness×springiness, which is the energy required to chew a solid sample to a steady state of swallowing [4]. TPA measurements were performed in at least 7 to 10 cores per sample.

### Warner-Bratzler shear force measurements

Instrumental tenderness was measured using the WBSF test, assessing the resistance to shear force cut in cooked meat as described before [26]. Samples were obtained by cutting 1.3 cm diameter cores from each steak parallel to the muscle fiber orientation and sheared once across the middle (in the direction perpendicular to the fibers) using a 1.016 mm WB probe in a TA.XT Plus Texture Analyzer (Stable Micro Systems Ltd, Surrey, UK). The highest peak in Newton (maximum shear force) was measured in 7 to 10 cores per sample.

To further analysis, meat samples were grouped according to their hardness values obtained by the WBSF test, considering the shear force threshold of 58.8 N suggested by Shackelford et al [27], which proposed that meat samples with shear force values lower than threshold could be deemed tender and a sample was deemed tough if its WBSF value was 58.8 N or greater. In this sense, samples with WBSF values below threshold were grouped in the Tender group (mean 54.98 N) and samples with higher values in the Tougher group (mean 74,91 N). This threshold value was chosen previously as the margin between tender and tough because the regression of trained sensory panel tenderness ratings on shear force indicates that a sample with a shear value of 58.8 N will be rated “slightly tender” on average.

### Scanning electron microscope

Samples, cut longwise to the muscle fibers, were fixed with Karnovsky’s solution (2.5% paraformaldehyde and 1.5% glutaraldehyde) in 0.1 M of dibasic phosphate (pH 7.2) at room temperature. Subsequently, the samples were rinsed twice with distilled water and dehydrated with increasing ethanol gradient solutions (e.g., 30%, 50%, 70%, 90%, and 100%) for 10 minutes in each solution at room temperature. The samples were then placed in 100% acetone for 10 min, dried with CO_2_ at the critical point condition (DCP-1 Critical Point Dryer; Denton Vacuum, Moorestown, NJ, USA), mounted in a holder with a two-sided adhesive tape, and coated with gold twice for 10 and 20 min. The ultrastructure was evaluated with the Phenom ProX scanning electron microscope (SEM; Thermofisher, Eindhovem, Netherlands) with an accelerating voltage of 5 kV and a working distance of 2 mm. SEM images were obtained at 1,000× magnification for fiber and 15,000× for sarcomere structure evaluation. Sarcomere length and muscle fiber diameter were calculated using the apparatus software.

### Raman spectroscopy

Before Raman spectroscopic measurements, subcutaneous fat and perimysium were removed in all meat samples. Experiments were conducted with a LabRAM HR Evolution confocal microscope (Horiba Scientific, Villeneuve d’Ascq, France) equipped with a 785 nm laser of 103 mW. All spectra were performed so that the incidence of the excitation laser beam was perpendicular to the direction of the muscle fibers. To improve the signal-to-noise ratio and to obtain the maximum exposure without saturation of the spectra, an integration time of 3 s and 40 accumulations were used, which is equivalent to a total measurement time of 15 min per loin. Raman spectra were preprocessed using Savitzky–Golay smoothing filter with a polynomial of 3rd order and baseline corrected using rubber band baseline correction (RBC) in LabSpec 6 software. All measurements were performed in triplicates to obtain a representative sample.

### Statistical analysis

The Student’s t-test was used for independent samples to compare Tender and Tougher samples using animals as the experimental unit. Means of quality parameters were compared using Infostat software [28]. For all assays, the level of significance was set at 0.05.

To determine whether Raman spectra could predict meat and quality attributes, principal component analysis (PCA) was applied with the RamanToolSet free software [29]. The PCA model was calculated using a *k*-fold (*k* = 3) systematic cross-validation procedure to enhance model optimization. Pearson correlation coefficients were measured using the preprocessed Raman spectra as independent variables (“predictors” or X variables) and the physicochemical values as dependent variables (Y variables).

## RESULTS AND DISCUSSION

### Physicochemical parameters of meat quality

Physicochemical parameters measured for meat quality characterization of Braford meat samples, such as hardness, cohesiveness, chewiness, WBSF, WHC, CL, IMF, fiber diameter, and sarcomere length, are listed in Table 1.

Hardness, WBSF, and cohesiveness values were higher in beef from tougher samples than that from tender ones (p< 0.0001, respectively), as was expected. Nevertheless, no differences in chewiness were observed between the groups. However, WHC and CL values were significantly different between groups, resulting in higher WHC and lower CL in the tougher meat samples compared to the tender ones with p<0.0001 and p = 0.0006, respectively. In addition, tender meat samples had a lower IMF content than the meat from the tougher group (p = 0.0174).

The data in Table 1 indicate that fiber diameter was similar between both groups (p = 0.1657). However, the Tougher group showed longer sarcomeres than the Tender samples (p<0.0001). The molecular architecture of the sarcomere as defined by the myosin filaments, the interaction with the actin filaments, and the boundaries formed by the Z-disks will subsequently influence basic meat quality traits such as tenderness and water-holding capacity [30].

**Figure 1:**
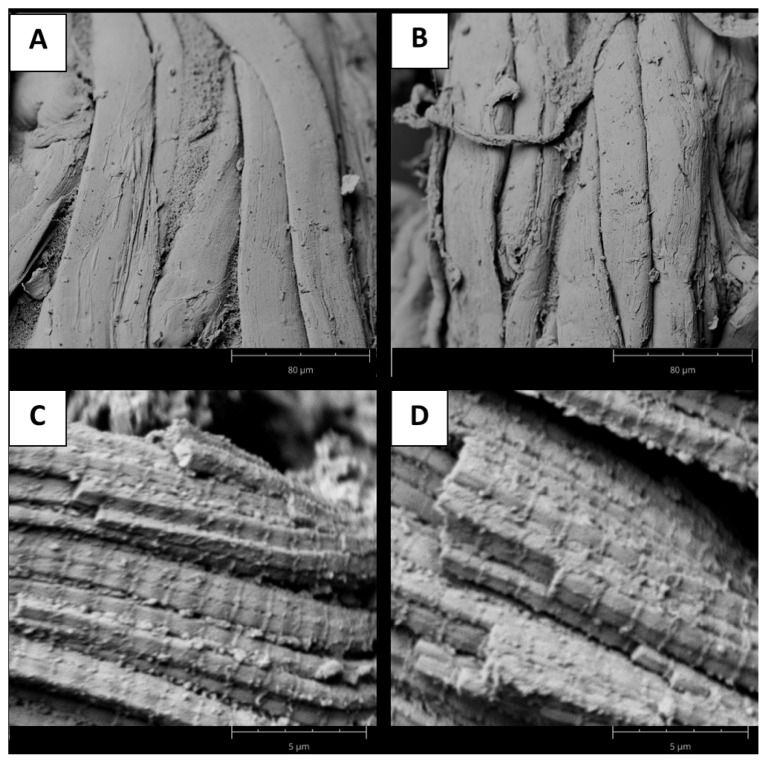
Microestructure of myofibrilar structure in beef samples. Scaning electron microscopic (SEM) images were obtained using 1,000× magnification for tough (A) and tender (B) samples, while for sarcomere structure a magnification of 15,000× was selected for tough (C) and tender (D) samples.

In this regard, SEM images confirmed that the myofibrillar structure of the meat was very regular in both groups, with neighboring myofibrils adhering to each other and noticeable sarcomeres and Z-discs (Figure 1). Previously, was suggested that differences between tough and tender samples were associated with structural changes within myofibrils [9], and several authors described this difference using relationships between sarcomere length and tenderness [31,32]. In addition, hardness evaluated as WBSF, was higher in muscles with sarcomere lengths between 1.60 and 1.70 μm, while they were lower in muscles with sarcomere lengths of 1.40 to 1.50 μm, as previously reported for lamb meat [32].

The release of water from the meat could also be explained based on structural changes within the sarcomere, by the involvement of the degree of overlap of myosin and actin filaments and the number of cross-links between them during the development of *rigor mortis*, since with increasing sarcomere length, the degree of overlap of myosin and actin filaments becomes smaller and, therefore, so does the repulsion between filaments [30]. In this regard, as in a previous report [33], we found here that meat with longer sarcomeres has a higher WHC value. Ertbjerg and Puolanne [30] explained that the reduction in water retention at shorter sarcomere lengths is due to the stronger attraction created by the greater number of cross-bridges, and shorter sarcomeres produce longer distances between longitudinal filaments and thus less electrostatic repulsion.

Intramuscular fat has an indirect relationship to meat tenderness since data in Table 1 indicate that the tender group showed lower IMF content than the tougher samples. This results are in concordance with previous works made in Braford cattle bred [26,34,35]. This result could be associated with a prevalent lower protease activity due to the presence of IMF, which results in a decrease in meat tenderness [36].

### Raman spectra of meat samples

**Figure 2:**
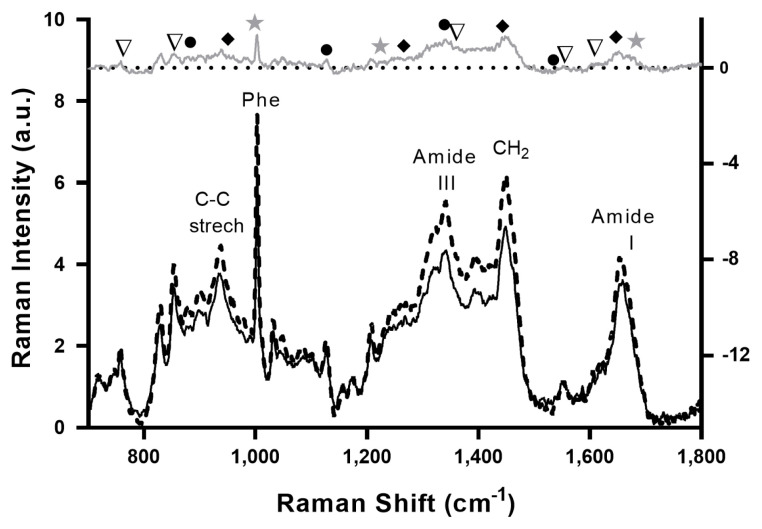
Averaged Raman spectra of the tenderest (black solid line) and the toughest (dashed black line) samples. The gray upper curve is the difference spectrum “tough minus tender”. Selected signals are indicated by symbols: β-shift proteins (star), α-helical proteins (diamond), myoglobin (circle), tryptophan, and tyrosine doublet (triangle).

Figure 2 shows the averaged Raman spectra of tender (black solid line) and tough (dashed black line) meat samples, with Raman signals typical of muscle tissue representing the major vibrations of amino acids chemical bonds, including tryptophan, tyrosine, and phenylalanine, as well as those protein backbone conformations and secondary structures [3]. The Raman spectrum of Tender and Tougher samples shows almost the same signal fingerprint, although slightly higher intensities were observed in the tougher than in the tender meat samples, in agreement with other studies [17,18].

Although it is not possible to assign with certainty any differential peak between the Raman spectra of beef and tender meat, a tentative interpretation of the spectra based on the evaluation of the “tough minus tender meat” difference spectrum (gray top spectrum in Figure 2) is plausible. The Raman band centered near 1,653 cm^−1^ represents the amide I band, which is an indicator of the overall concentration of proteins [11]. In turn, the vibrations centered near 1,250 cm^−1^ represent the amide III band which is sensitive to secondary and tertiary structures of proteins [37]. The band at 1,446 cm^−1^ is assigned to the −CH_2_ scissor, which decreases with increasing hydrophobicity in the molecular environment. The band at 1,003 cm^−1^ is assigned to phenylalanine ring stretching, while bands centered around 900 and 1,130 cm^−1^ are assigned to stretching modes of C-C and N from lipids and proteins, respectively [35]. Previously was demonstrated that the level of saturated fats increases with increasing levels of IMF [38], and this contributes to increase C-C stretching vibrations (1,020 to 1,130 cm^−1^) and CH_2_ twists (1,300 cm^−1^) [15], explaining differences observed in these regions in Raman spectra between toughest (with higher IMF) and tender meat.

Typical signals of meat which can be attributed to α-helical proteins are increased in tougher samples as indicated by peaks at 1,650, 1,447, 1,274, and 935 cm^−1^. Furthermore, signals of myoglobin (1,540, 1,340, 1,126, and 885 cm^−1^) and the aromatic amino acid side chains, notably tryptophan (1,557, 1,360, and 762 cm^−1^) and tyrosine (1,610 and 855 cm^−1^) are also enhanced in this group. Previously was reported that the tyrosine doublet signal weakened in tough meat is the direct reflection of less proteolysis [35]. Moreover, the tougher meat contains a higher proportion of β-sheet structure (1,665, 1,235, 1,006 cm^−1^) than the tender meat [3]. The present results agree with these findings since tougher samples showed a higher α-helical to the β-sheet ratio in comparison to the tender meat. In this regard, when denaturation takes place the protein structures convert from α-helical into β-sheet or random coils, and thus, the Raman signal corresponding to α-helical becomes weaker [14].

The higher intensities observed in Raman spectrum of tougher samples can be understood by differences in packing of proteins as the Raman intensity is proportional to the number of scattering molecules; by an increase of the polarizability of the molecules due to changes in the surrounding or by a general change of the tissue’s optical properties leading to increased backscattering [17].

### Principal component analysis

**Figure 3:**
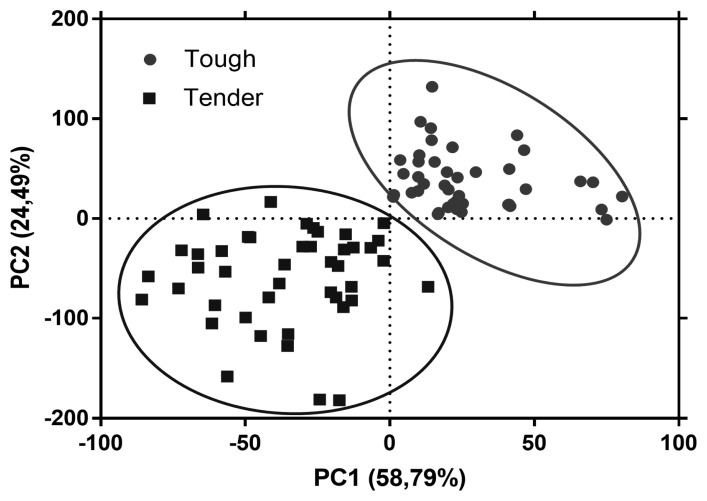
Principal component analysis (PCA) scores plot showing separation of tough (circle) and tender (square) meat samples in the principal component 1 (PC1) and 2 (PC2).

**Figure 4:**
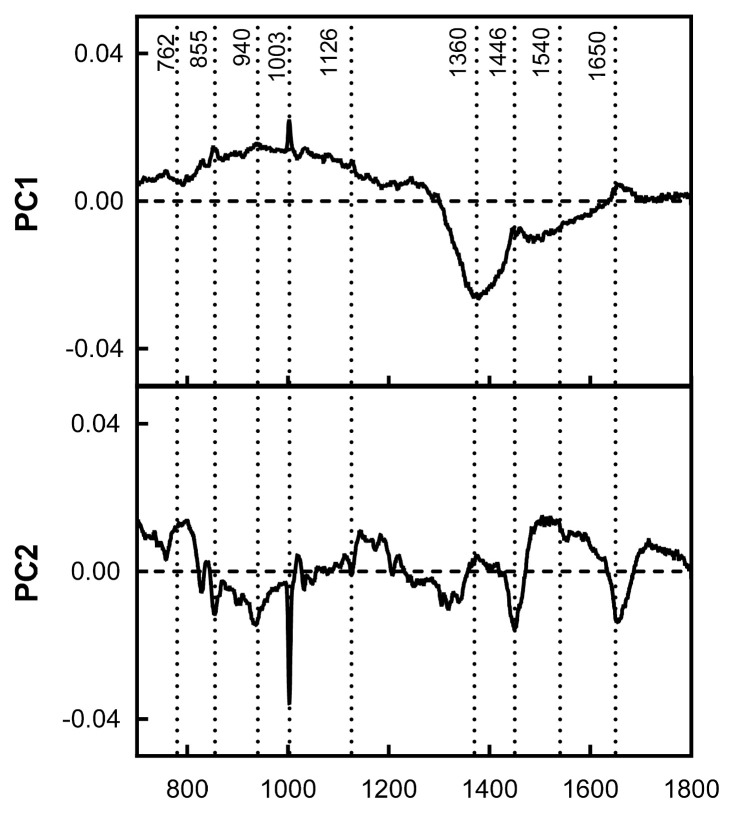
Principal component analysis (PCA) loadings plot with Raman spectral signatures associated with variation in tough and tender samples in component 1 (PC1) and 2 (PC2).

Raman fingerprinting can be used to construct chemometric models to classify and/or differentiate meat samples with distinct properties [39]. The first two principal components explained 83.28% of the variance between both groups of meat samples. The plot of the PCA scores shows that the tougher meat samples clustered in the positive space of PC1 and PC2, while the tender samples clustered in the negative space of PC1 and PC2 (Figure 3). The loadings plot in Figure 4 shows the Raman signatures responsible for the PCA discrimination. The Raman signals at 762, 855, 940, 1,003, 1,126, 1,360, 144, 1,540, 1,650 cm^−1^ were associated with the highest variance. Though meat samples where PC1 and PC2 clustered in positive space were associated with phenylalanine (1,003 cm^−1^), tryptophan (762 and 855 cm^−1^), and myoglobin (1,126 and 1,540 cm^−1^), which are stronger bands in these samples. Tender meat samples clustered in negative spaces PC1 and PC2 were associated with phenylalanine, α-helix, CH2-scissors (940 and 1,446 cm^−1^), and amide I (1,650 cm^−1^).

**Figure 5:**
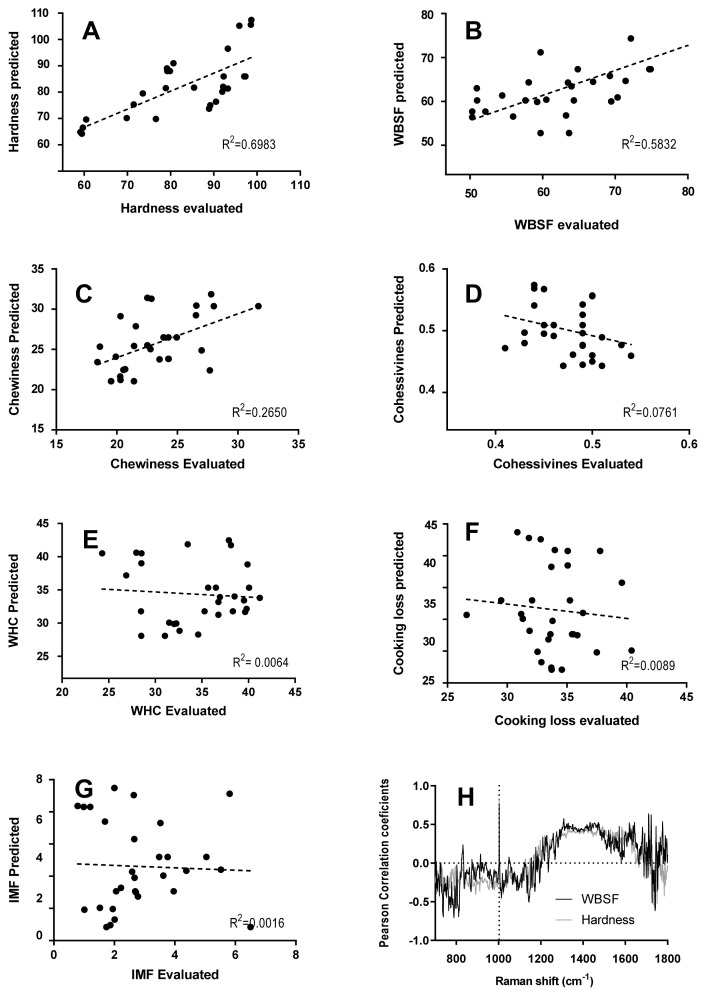
Relation between evaluated and predicted physical measurements using linear regression model. The relationship between evaluated and predicted physical measurements using linear regression model. R^2^ with a 95% confidence interval (dotted line), (A) Hardness, (B) Warner-Bratzler shear force (WBSF), (C) Chewiness, (D) Cohesiveness, (E) Water holding capacity (WHC), (F) Cooking loss (CL), (G) Intramuscular fat content (IMF), and (H) Pearson correlation coefficients (r) between Raman spectral data and hardness (gray) and WBSF (black) values; n = 30 samples.

Figure 5 shows the correlation between meat quality traits obtained from Raman spectra and physicochemical measurements. The correlation coefficient is the unit of measurement used to calculate the intensity in the linear relationship between the variables involved. If the coefficient value lies between ±0.50 and ±1 is a strong correlation; if the value lies between ±0.30 and ±0.49 is a moderate correlation and the value lies below ±0.29, denotes a weak or no correlation. The analysis performed results in a strong correlation for hardness (R^2^ = 0.6983) and WBSF (R^2^ = 0.5832). And moderate or weak correlation for chewiness (R^2^ = 0.2650), cohesiveness (R^2^ = 0.0761), WHC (R^2^ = 0.0064), CL (R^2^ = 0.0089), and IMF (R^2^ = 0.0016).

Promising results were reported with Raman spectroscopy in lamb and pork meat. Schmidt et al [40] reported higher coefficients of determination (R^2^) of 0.72 and 0.86 when predicting shear force values from carcasses in *longissimus thoracis* and *longissimus lumborum* lamb muscles, respectively. Moreover, Beattie et al [37] reported R^2^ of 0.77 and 0.75 for predicting shear force in porcine *longissimus* and silverside bovine muscle, respectively. Nevertheless, Fowler et al [14] and Fowler et al [15] reported lower coefficient of determination with Raman spectra and shear force value in *semimebranosus* and *longissimus lumborum* (0.27 and 0.06, respectively) lamb muscle, and Santos et al [13] showed that sensory tenderness and shear force values in porcine muscle are weakly correlated (R^2^ = 0.2).

Our results gave a relatively good accuracy for predicting hardness (by TPA and WBSF) in comparison to previous Raman studies with different bovine muscles. For instance, Beattie et al [3] reported higher coefficients (R^2^ = 0.75) in silverside muscle, Chen et al [19] obtained R^2^ of 0.81 for tenderness in *longissimus dorsi* muscle from bulls, and Nian et al [41] found an R^2^ of 0.75 in predicting WBSF of young dairy bull beef. Nevertheless, Bauer et al [17] and Fowler et al [16] described lower R^2^ values (0.33 and 0.11) in the *gluteus medius* and *longissimus lumborum* muscles, respectively.

Nevertheless, our results showed a poor correlation with IMF (R^2^ = 0.0016). However, IMF was previously predicted with higher R^2^ in pork muscles and beef (R^2^ = 0.73 and 0.89, respectively) [18], suggesting that the amide I and III regions could predict this attribute due to the contribution from lipids including C-C stretching (1,660 to 1,640 cm^−1^), −CH_2_ twisting (1,300 cm^−1^), and C–H deformations (1,270 cm^−1^).

Cama-Moncunill et al [18] also evaluated samples with different cooking loss values and reported that the Raman spectra recorded from samples with the lowest and highest cook-loss displayed little differences, which for the most part were observed at the region between 1,000 and 870 cm^−1^ where samples with lowest cook-loss presented slightly higher intensity values. Compared to the present work, Cama-Moncunill et al [18] showed in a sample set performed with that aim that the cooking loss model exhibited high predictive ability (R^2^ = 0.67).

Furthermore, Pearson correlation coefficients between hardness and WBSF values and Raman intensity at each wavenumber of all spectra are shown in Figure 5. Pearson correlation coefficient at the signal pattern in the 1,250 to 1,650 cm^−1^ was moderately correlated and at 1,003 cm^−1^ was highly correlated to hardness (r = 0.84) and WBSF (r = 0.79), explaining this physical measured parameter. As was mentioned above, the signal pattern in the 1,250 to 1,600 cm^−1^ includes some signals attributed to α-helical (1,650, 1,447, 1,274 cm^−1^) and β-sheet structure (1,665, 1,235 cm^−1^), associated with meat tenderness. Moreover, Beattie et al [37] found that the peak of the aromatic amino acids such as phenylalanine contributed to the correlation with shear force. The phenylalanine band at 1,003 cm^−1^ is insensitive to the chemical and physical environment within the sample. This signal is sensitive to the relative abundance of this amino acid and so if the protein composition (or abundance) changes, the relative height of the phenylalanine band will change accordingly.

Although Raman spectroscopy is a robust and reproducible technique, it should be kept in mind that the prediction of meat quality by Raman spectroscopy is strongly influenced by several experimental issues, e.g., animal breed, muscle type, Raman device (handheld or benchtop), and acquisition characteristics (laser wavelength, integration time, accumulation of spectra, etc.), as well as the type of chemometric analysis selected, as can be seen in the literature. Therefore, Raman users should be aware of the above issues for the correct use of the technique in meat quality control.

## CONCLUSION

To our knowledge, this is the first report that shows the capability of Raman spectroscopy in combination with PCA chemometric analysis to predict hardness (R^2^ = 0.698) and WBSF (R^2^ = 0.583) values in Braford *longissimus thoracis et lumborum* muscle. Furthermore, Raman spectroscopy is an adequate technique to identify Braford beef samples with differential tenderness attributes. Although the Raman pattern was similar in both tender and tougher meat samples, the signal intensity was higher in the latter, particularly for the peak due to phenylalanine at 1,003 cm^−1^ and in the Amide III band, with a change in the ratio between the α-helical and β-sheet, suggesting that both the relative composition of the residues and the secondary structure of the meat proteins are affected. The high value of Pearson’s correlation coefficient of hardness parameter with the Raman signal at 1,003 cm^−1^, assigned to the symmetric vibration frequency of the phenylalanine ring, suggests that the content of this amino acid explains the differences between groups of samples. Results suggest that this technique could be a non-invasive and non-destructive method for the screening of individuals with tender meat. The meat industry would benefit from the ability to select animals that are superior and consistent in meat quality traits.

## Figures and Tables

**Table 1:** Mean and standard deviation of the values of physical measurements of tender and tough meat samples

Items	Tender meat	Tougher meat	p-value
Fibre diameter (μm)	46.92±10.87	44.12±7.96	0.1657
Sarcomere length (μm)	1.41±0.19	1.71±0.22	<0.0001
IMF (%)	2.22±1.44	3.47±1.24	0.0174
WHC (%)	31.02±3.52	37.75±2.53	<0.0001
CL (%)	35.51±2.29	32.34±2.48	0.0006
WBSF (N)	54.98±4.23	74.91±10.60	<0.0001
Hardness (N)	70.51±7.54	93.89±8.09	<0.0001
Cohesiveness	0.50±0.02	0.46±0.03	<0.0001
Chewiness	24.28±3.60	22.33±2.47	0.0950

IMF, intramuscular fat; WHC, water holding capacity; CL, cooking loss; WBSF, Warner-Bratzler shear force.
